# The Training Setting as a Social and Liminal Space for Professional Hybridization

**DOI:** 10.3389/fpsyg.2021.804008

**Published:** 2021-12-24

**Authors:** Giuseppe Scaratti, Ezio Fregnan, Silvia Ivaldi

**Affiliations:** ^1^Department of Social and Human Sciences, University of Bergamo, Bergamo, Italy; ^2^Department of Psychology, Catholic University of the Sacred Heart, Milan, Italy

**Keywords:** liminality, liminal space, hybridization, narrative space, training

## Abstract

This article addresses the liminality concept as a way to explore a particular group context, relating to a training setting intended as a liminal space, and to highlight its potential to trigger evolutionary personal and organizational identity trajectories. Dealing with a contemporary uncertain, volatile, and ambiguous organizational scenario, people are asked for consistent and quick professional hybridization processes. This article refers to a case study related to an action research process aimed at a cultural transformation and nurturing organizational learning inside an extra-hospital Rehabilitation Center, challenged by a strong organizational reconfiguration and the creation of new functions and roles, among which the one *coordinator*, responsible for the operational activity to be managed within the units of the organizational context. This article also highlights both the main features that characterize a training setting as a liminal space and identifies the possible plots of professional hybridization paths that a training group as a liminal space can trigger and develop.

## Introduction: Adhocratic Organizations and Professional Hybridization

The evolution toward pluralistic organizational scenarios, which are characterized by an understanding of different goals and interests among the groups that are internal and external to organizations, requires the activation of an agile working mode (Harris, 2015, 2016; Fregnan et al., 2020; Ivaldi et al., 2021) and an adhocratic approach to organizational architecture (Mintzberg, 2009, 2012).

People and organizations are called to a challenging learning path, seeking to cope with ever more demanding complex situations and environments, to solicit the relations between identity (professional and organizational), knowledge, and practice. They are facing to a runaway object (Engeström, 2008), which is progressively defined, shaped, and shifting, as the unfolding progress of its achievement is emergent due to a multi-faceted, complex, and often contradictory combination of participants, communities, instruments, rules, and the division of labor.

Hence, the solicitation of consistent and quick professional hybridization processes, dealing with contradictions in the daily organizational them (Ivaldi and Scaratti, 2020), experience and the simultaneous need to make sense of them, seek new possibilities of action related to the unfolding object-oriented activity. The challenge is about opening a portal (Engeström, 2020) to enhance a new and not yet explored a way of thinking about something, seeing differently and fitting action, sense-making, future-oriented vision, and conflicting dynamics.

Tackling of organizational contexts characterized by meaningless and uncertainty, in which people have to deal with unforeseen problems, criticalities, and trouble situations, requires the adoption of an anchoring forward step perspective (Sannino and Laitinen, 2015). It entails coping with transformative actions and sense-making processes connected to the experience of stepping into the unknown and improvising paths for new actions and meanings.

This article aims to explore how much a particular group context related to a training setting intended as a liminal space supports and facilitates professional hybridization processes within an organizational context undergoing restructuring and transformation. To this end, a training experience is presented within a research-action path, developed in a health and social center, in which the identity transition path of some key figures was monitored: the coordinators of operational units. The goal is to grasp how an appropriately configured liminal training space has facilitated the evolution of identity trajectories, allowing processes of adaptation in one’s professional, and organizational identity.

There are two research questions that the article intends to develop:

- What are the pivotal features that characterize a training setting as a liminal space?
- What are the possible plots of professional hybridization that a training group as a liminal space can trigger and develop?

To answer these research questions, we refer to a socio-constructionist approach, both at the theoretical and practical level, to gather a narrative (Ibarra, 1999; Ibarra and Barbulescu, 2010) and conversational (Zucchermaglio, 2013) material within the training context and to be also used as a space for a discussion and collective validation of the empirical evidence collected, thus enhancing the concrete experiences of practitioners involved and giving space to their voices as practical authors (Cunliffe, 2001).

This article unfolds as follows: we begin by highlighting the concept of liminality and its connection with the challenging trajectory of professional hybridization. Thereafter, we address the pivotal aspects of a training setting as a liminal space and conceive it as learning by experience and transformative path (Mezirow, 2000; Scaratti, 2017). Then, we point out a specific training experience related to a specific organizational context, underline the methodological perspective adopted for gathering empirical data from the field, and present the main findings connected to the experience of people involved. Finally, we conclude by addressing some transversal dimensions at stake and by suggesting hints for further research.

## Liminality, Professional Hybridization, and Training Setting as a Liminal Space

Beech (2011) points out how the notion of liminality relates to social anthropology (Turner, 1984) as a ritual of transition (Van Gennep, 1909/1960) that entails a time and space “betwixt and between” (Turner, 1966; Chreim, 2002). Such a time and space condition in the ancient societies was ritualistically defined while nowadays it is more blurred and nuanced; however, maintaining the meaning of a liminal process through which a position of ambiguity and uncertainty is crossed. At stake is the intersection between persons and social structures (Ybema et al., 2009), dealing with the rituals of transition that accompany the individual/group’s change of social status, described by Van Gennep (1909/1960) as separation (divestiture), transition (liminality), and incorporation (investiture). The reconstruction of identity is required to cross a *social limbo* (Turner, 1982; Czarniawska and Mazza, 2003) in which the rules of daily reality are suspended, as at theater (Turner, 1984), facing the disruption to the sense of self (Noble and Walker, 1997).

We follow Beech (2011) in pointing out the potential of liminality conceptualization for using it in the organizational field as the changeful nature, the multiple meanings, and the psychological consequences to tackle with the “instabilities in the social context, the ongoing ambiguity, and multiplicity of meanings, the lack of resolution (or aggregation), and the substitutability of the liminar…as a longitudinal experience of ambiguity and in-betweenness within a changeful context” (Beech, 2011, p. 288). Indeed, practitioners who are engaged in actual organizational contexts are asked to explore “processes of learning from the fields, connecting action and thought and trying to open new visions not yet available for transforming and improving their daily practices” (Scaratti and Ivaldi, 2021, p. 5). In a Volatility, Uncertainty, Complexity, and Ambiguity (VUCA) scenario (Bennett and Lemoine, 2014), they often work on more than one object simultaneously, experiencing plural and different approaches to the same workplace task and living a fractional (Law, 2002) relationship with the organizational processes of differentiation and integration, which underpin the concept of organization as organizing (Czarniawska, 2008).

Hence, the challenge of professional hybridization processes is related both to hybrid managers who are pivotal in the dynamics of organizational change and to all professionals who require to view organizational issues through plural and often divergent windows and perspectives (Ibarra, 1999; Ferlie et al., 2005; Chreim et al., 2007; Ellis and Ybema, 2010; Ibarra and Barbulescu, 2010; Beech, 2011; Ivaldi and Scaratti, 2019; Engeström, 2020). At stake is the possibility to address the multiple dimensions of precariousness and lack of knowledge to be faced, looking progressively for exit routes (search actions, taking over actions, and breaking out actions: Sannino and Laitinen, 2015), using transitory and actionable knowledge (Shotter, 2008, 2010) to help a sustainable positioning in uncertainty, moving into the trouble area, and trying to get out. Professionals and practitioners are asked to rethink their working practice and to address the intersection between activity, experience, and context (Green, 2009).

The *activity* is a configuration of specific actions and operations-oriented to an object and endowed with its own structure, which is defined through historical, social, and cultural processes and is based on material and immaterial conditions that allow its realization (Engeström, 1999). Each activity system is crossed by tensions and contradictions that mark its evolutionary process, and the possibilities of transformation, passing through constant learning processes (expansive learning) that constantly configure and reconfigure tools, rules, the division of labor, languages in use, participants, and object of the activities in which people are involved. Professional practice has to do with positioning oneself within these systems of activity and with the possibility of expressing one’s transformative action in them.

The *experience* refers to our life as it is characterized by different levels of awareness, memory, orientation to the future, the relationship with space, time, emotions, feelings, affections, sensoriality, and corporeality. Professional practice is embodied and deeply inserted in an experiential texture that connotes and expresses it, crosses it, and innervates it.

The *context* refers to the material and immaterial background that constitutes the symbolic and operational plot within which our concrete work is located. Professional practice cannot be conceived if not situated in contexts in their extensional configuration (material, logistic, and structural) and intentional (meanings, languages, attributions, etc.), which settle in tacit knowledge, in routine, in widespread implicit uses, in meanings taken for granted, and conveyed through rules and rituals. Rethinking working practice solicits the transformation of professional identity, which was reinterpreted and understood in the process of tuning between the worker and context through negotiations and joint constructions of culturally shared meanings.

Referring to Bruner (1990, 1995), we could advocate a social and distributed professional identity, meaning that the contextual dimension and the constant dynamic of shared construction of knowledge were in constitutive dialogue with the social and cultural landscape that characterizes the human being. The process of professional hybridization requires a hermeneutic, negotiation, and conversational disposition that the practitioner establishes in constant transactions with others and with the world, learning to relate to the events he/she encounters and to position him/herself appropriately in the relational, professional, and working dynamics that he/she lives and goes through. Such a stance entails the intertwined declination of three relevant dimensions: phronesis, praxis, and aporia (Green, 2009).

The concept of *phronesis* addresses a process incorporated in constant interaction with contexts, practices, experiences, common sense, intuition, and judgment, in which what is at stake is the ability to configure action-oriented knowledge, especially in situations in which neither already acquired knowledge nor scientific references are available (Dunne, 2005).

The principle of *praxis* refers to the concept of “good” action, oriented toward good, opening up the theme of the connection between our actions and the ethical dimensions connected to them, both at the level of personal choices and at the collective level. The possibilities of pursuing a good job, the capability of being done well, and the capability of generating common value and personal pleasure/satisfaction are the essential references for a professional positive identity.

Finally, the notion of *aporia* refers to the confrontation with inevitable paradoxes and contradictions that require the assumption of our responsibility, our positioning, and making decisions in a scenario characterized by high ambiguity and changeability. These are increasingly frequent situations, in which the professional is required to act even if it is not clear to him/her what will happen next and has to improvise (Polkinghorne, 1997), dealing with a structural uncertainty.

Facing such challenges requires the activation of liminal spaces (Thomas and Linstead, 2002; Beech, 2011), which are socially connoted and capable of allowing the focus of one’s own identity trajectory, avoiding losing the plot (Thomas and Linstead, 2002), as well as the crossing of organizational boundaries and the mobility between different social and professional groups (Czarniawska and Mazza, 2003).

We specifically address a particular configuration of the training setting, focusing on the lived and contextualized experience of the professionals, as a liminal space for enhancing the generative and developmental exploration of the relationship between professionals and organizations. As a social context, such a training setting conveys the possibility to cope up with different interpretations, plural personal/professional narratives and trajectories, multiple and often contradictory ways of understanding the same work or organizational place (Scaratti, 1998). The training setting itself becomes a liminal space, in which a provisional separation is enacted from the activity system, creating the conditions of an in-between zone among the *here and now* of the training setting and the *there and then* of the daily course of action in which people are involved (Scaratti, 2010, 2017). Liminality is configurated with blurring and merging of distinctions (Czarniawska and Mazza, 2003), seeking to achieve new perspectives as well as to improve transformations triggered by the training process (Scaratti and Kaneklin, 2010).

Following a socio-constructionist approach, such a training setting offers proper conditions for a dialogue between self and social identity (Watson, 2009), prompting the participants to adopt both a centripetal and centrifugal perspective (Bakhtin, 1981): the first relies on what people internalize from the society and external sources while the second is about what people externalize from their own experience. Both the movements facilitate the enhancement of liminal practices as *experimentation, reflection, and recognition* (Beech, 2011). The practice of *experimentation* means that through situated exchanges, participants share the experience to shape and reshape their professional and organizational identity, coping up with individual differences, contextualized multiple voices, and intertwined practices.

The practice of *reflection* conveys a reflexive work upon subjective, intersubjective, and institutional levels, addressing the liminality to the day-by-day lived experiences, reviewing and questioning history, language, discourses, and actions.

The practice of *recognition* improves the personal and social process of the acknowledgment of own identity unfolding accomplishment, giving the participants the opportunity to meet plural narrative and discursive trajectories as the training setting becomes the loci of the constitutive place of professional and organizational identity.

Hence, these liminal practices may be addressed in an *organizational narrative space* (Scaratti, 2021), where linguistic accounts (stories and tales, discourses, conversations, documents, and contextual emergent processes) are the connecting keys of intertwined and unfolding plots of actions, people, and events. Approaching cultures, languages, and representations with which participants see, look at, and organize their daily experience generates a meeting place between the different participants who reciprocally exchange reconstructions and stories, to shape an organizational knowledge that has an impact both in professional trajectories and in the evolution of the system of activity at stake (Scaratti et al., 2009).

Such a liminal training setting as a shared workspace becomes itself an ongoing narrative, which is defined by the negotiations and constructions and by the agreements on the realistic sustainability of the process agreed by the participants. It requires a desire for mutual listening, the willingness to let oneself go to imaginations and openings, which postulates a context of sufficient serenity and investment as well as the availability and sustainability of the actors to express and socialize their accountabilities relating to events and situations of the common organizational experience. Organizational actors also potentially become the authors of a possible and unpredictable renewed narration of the pact that binds them and of the reformulation, acceptance, or rejection of agreements on their future history and professional path (Cunliffe and Scaratti, 2017).

In the next section we point out the case and the organizational context in which the described liminal training setting was realized, highlight the methodology adopted to gather knowledge, and describe the main findings acquired through its use.

## The Context as a Place of Organizational Restructuration, Transformational Change, and Professional Challenge

The organizational context to which we refer is a private non-profit health service, born in 1966 as a “Psycho-Pedagogical Medical Center,” and currently configured as an Extra-Hospital Rehabilitation Center, following an accreditation provision required by Italian national policies and laws. The Center operates in northern Italy, dealing with children and young people suffering from multiple pathologies: neurological pathology from central and peripheral neurological damage; psychic disorders; autism spectrum disorder; and behavioral disorders associated with intellectual disability.

The Center offers residential, daytime, outpatient, and clinical interventions, in line with the needs of the patient and his family. With a total of 300 employees, 120 places are offered for resident patients, 60 places are offered for daily service, and about 150 places are offered for outpatient services.

The mission of the Center is inspired by the typical Christian values of its founder, providing care and cure to young guests, responding to their specific needs, both clinical and social.

The pivotal activity of the center is the residential service. It is an extensive treatment in which the patient’s living environment and the quality of relationships with the clinical education team and peers are the essences of the multidisciplinary and integrated therapeutic rehabilitation project. Such a therapeutic approach asks for a high level of emotional holding (Winnicott, 1964) and a relational style to be adopted as appropriate to the particular clinical characteristics of the users. The needs of the patient and his family are the focal points of the health assessment followed by the design of specific treatments.

The daytime service is configured as an intermediate reception structure, designed to support the suffering family unit, thereby allowing the guest to continue living in their own home. It is an intervention with therapeutic, rehabilitative, and educational–occupational functions aimed at children with neuropsychiatric and neuromotor disabilities; the “taking charge” is flexible and allows to safeguard the needs and priorities of the user, including, for example, school attendance.

The outpatient clinic service deals with neurological and psychiatric diseases in developmental age such as epilepsy, infantile cerebral palsy, psychomotor disability, “intellectual disability, autism, malformed syndromes, movement disorders, neuromuscular diseases, neuropsychological pathologies related to language and learning disorders, relationship and behavioral difficulties, and minor mental disorders.”

A complex organizational chart of 11 Operative Units, divided into specific fields of intervention (Autism Unit, Serious Cerebrovascular Unit, Psycho-Organic, Intellectual and Relational Disability Unit), involves a plurality of professional groups and disciplines, with practitioners devoted to different functions, tasks, and pathways (medical, nursing, social, physiotherapy, psychotherapy, administrative, economic, logistic, etc.). The organizational daily process entails a wide variety of professional families, matching long-lasting belonging practitioners with new comers and novices (with different training, specializations, and subcultures), plural structural and material spaces (most of the units are located in the principal building of the center, while others have a peripheral location), and different conditions and histories (due to the geographical location of the center, across different regional borders and related institutional rules and regulations).

After the death of a religious founder, the Center is managed by a President and a Management Board, composed of a General Director, a Health Director and Vice Director, HRM, Economics Manager, and Logistic Manager. Each is responsible for coordinating the activities across each function: managing employees and practitioners with different professional and job profiles and organizational levels, i.e., clinicians (doctors, nurses, psychiatrics, and psychotherapists) and professionals (educators, auxiliaries, and administrators).

In Spring 2015, the Center’s General Director asked one of the authors, as an academic consultant, for a training intervention related to the residential and semi-residential service (initial participants: the clinicians).

At the beginning of the 2nd year of training, the clinicians proposed spreading the training to all the practitioners in their units. The Management Board approved the project and called for an action research process aimed at a cultural transformation and at nurturing organizational learning widely (Cunliffe et al., 2019). The goal was to develop and enhance a common perception and representation of transversal issues related to organizational processes, conflictual dynamics, and the problems within the daily integration processes between plural and diverse professionals, trying to identify some critical issues and opening interventions of sustainable transformation. At stake were specific issues related to the work experience of the people involved; the search for transformative actions; the possibility of improving common knowledge connected to new meanings; and sense-making paths through a reflexive process of expansive learning.

It is important to underline as relevant features the aspects of organizational restructuring and transformation triggered by the action research process, which mobilized energies and resources, involving various professional figures and generating turbulence and dynamics of conflict about the problems to be faced, sources of uncertainty and insecurity. The training setting as a liminal space is specifically suitable to address such transformative situations, in which practitioners are required/pressed to rethink and reshape their professional plot, interpreting the blurred boundaries they have to cope not only as of the possible cause of personal and organizational opacity, rather also as an opportunity to cross through the social construction of their unfolding identity.

Within the context described, one of the organizational reconfiguration interventions was the creation of the *role of coordinator*, responsible for the operational activity to be managed within the aforementioned units. Alongside the clinical and therapeutic responsibilities assigned to various figures and levels (medical director, psychologists, neuropsychiatrists, and nurses), management supervision of each individual unit is in fact requested. This assignment requires complex attention to the multiple work processes that mark the daily life of the units, from work turnover to the verification of the implementation of scheduled activities, to internal and external communication with other figures and stakeholders, up to the connection between the various professionals and teams. These are middle manager figures with hinge and connection functions between the strategic indications and objectives to be pursued and their translation into daily operational practice. The choice of the managerial board for the assignment of this role was to invest in the figures of senior educators already working in the units, thus valorizing their knowledge of the contexts and their professional experience, instead of turning to resources outside the organization.

Such an organizational restructuring challenged the identity trajectory of many educators, both triggering competitive dynamics and expectations and generating fear and ambiguity in relation to a not yet defined role to be taken.

Hence, the opportunity to provide a training setting as a social and liminal context for shaping and acknowledging common meanings and facing the potential disruptive solicitations to the own professional and organizational identity. They had to face a new phase of their professional trajectory, crossing a period of ambiguity as a limbo, coping up with a disruption of their traditional identity and tackling with the shaping of a new experience, practicing, acknowledging, and reflexing on their new role.

Within the training setting, the incoming coordinators questioned the evolution of the coordination role in the light of experiences of consolidation and comparison between the different professional families, units, and structures to which they belong. The multiple nature of the role and the need to manage different levels, implications, and tasks guided the reflection of the group in comparison with regulatory constraints, contextual and organizational criticalities, individual, group, and organizational resources, and possible levers. The management of their co-built “mobility” and “hybridization” was introduced as part of the possibility of experimenting with a way of being in the role that can be experienced in a positive and proactive way, despite uncertainties and contradictions, adopting a sustainable way from material and relational point of view, in terms of opportunities and not only of constraints.

### Methodological Aspects

We adopted a methodological approach to gathering knowledge that is strictly related to the coordinators’ experience and situated in their specific working contexts. Following a socio-constructivist epistemological orientation, we actively involved coordinators to co-construct a social text (Chia, 1996), getting close to their daily experience as middle managers. In the first step of the training setting, we negotiated with the coordinators the opportunity to provide narratives and tales of their routed practice (Ibarra, 1999; Ibarra and Barbulescu, 2010), at-home ethnographical accounts (Alvesson, 2009), organizational documents, portraits of organizational events, and processes, reported discourses. In this way, we gathered a vast array of qualitative empirical data, upon which we prompted the discussion related to how they were shaping their social and organizational reality, discussing, constituting, and reconstituting their role identity.

Topics such as turnover, or the reception of new hires and other similar, have opened a space for a discussion on paradigms and models, approaches, and artifacts in use and potential, highlighting representations and interpretations about the nature of the work within the center, its constrains and potentials, reflecting its lights, and shadows in a balanced way.

The training setting involved 16 coordinators of different organizational units for a period of 6 months after their formal role’s acceptance, from June to November 2017, providing eight meetings of half a day for a total of 32 h, approximately one for each month. The training objectives were to achieve a convergent interpretation of the common role, sharing the main contradictions to be faced and developing sustainable trajectories for enhancing the hybridization professional process (the educators became coordinators as well as other middle managers related to different professions).

The possibility of discussing and sharing the aspects of sustainability of everyday working life and service management, touching on issues of culture and professional identity, practices, contradictions, and inertia of organizational life, provided by the multiple accounts gathered by the coordinators, generated a vast array of linguistic, discursive, and conversational materials. The liminal training setting became itself a source of individual and collective discourse, comments, social constructions of commonly shared meanings (Zucchermaglio, 2013), giving coordinators voice for becoming practical authors of their organizational context and conveying authenticity and depth to the training process.

For the data collection, we requested informed consent from participants for recording the training meetings: they accepted the audio recording while disagreeing with the videotaping. The audio-recorded conversational data were transcribed, following Jefferson’s approach, and compared with the field notes of one of the two researchers involved in the meetings (one with the role of a conversational facilitator, the other as a participant-observer in the interactive process, tackling ethnographical notes). Since the data have been collected in the local language and subsequently translated into English, we worked as follows: first, we realized a translation done by a native speaker, and then we asked the other two different native speakers to validate the translation and to select the excerpts that achieved a convergent assessment rating of appropriateness.

The researchers analyzed the data coming from the interactive sessions, seeking to view the conversational text as a culturally situated practice, highlighting the historical and situated nature of the specific conversational social context (Drew and Heritage, 1992).

Both the main plots and the excerpts, highlighted in section “Key Findings: Crossing the Social Limbo of the Liminal Space,” were proposed by the researchers and discussed in the final meeting with the participants, who validated the proposed findings through a social discussion, at the end acknowledging and approving the emergent data. In this way, we valorized the relevance of professional groups and communities, sharing a sense-making process and highlighting personal and organizational trajectories to be followed.

In section “Key Findings: Crossing the Social Limbo of the Liminal Space,” we address the attention to the main plots generated by the training setting interpreted as a liminal space, dealing with a content analysis related to some excerpts picked up from a large basket of the available conversational data collected from the earlier depicted interactive setting.

## Key Findings: Crossing the Social Limbo of the Liminal Space

The exchange and conversational discussion between the coordinators within the training setting have gradually brought out some plots of meaning as shared interpretations in dealing with common role interpretations.

The first plot is about *dwelling the boundaries between roles*: it refers to sustainability as a delicate issue in the management of different roles by the same professional. Transversality and adaptability are required with respect to often unclear perimeters and increasingly blurred identity and organizational boundaries, as in the following conversational excerpt [related to the second training meeting after 1 month from the role’s acceptance by coordinators (in the excerpts C1, C2,…refer to the different coordinators)]:

(C1): It is difficult to balance an educator/coordinator figure. For M. is evident … she is the coordinator, maybe she intervenes in an emergency but only when there is a need. We are coordinators 24 h a day and take time away from both the educational and the coordination part.
(C2): It is the hot potato of coordination; it is difficult to continue to work as an educator …
(C1): Sometimes you should do your work at home, and it doesn’t seem right; I went to Lidl (a brand of a supermarket) to work on turnover…really, they often call you while you are in the supermarket. Each unit has its own critical issues, our kids cannot rest easy, and we cannot break away without putting their colleagues in crisis.
(C3): I find it difficult to interface… as coordinator I should interface with all the realities of the center, but it happens to see people sporadically, to speak little, it is difficult to hear each other. It becomes almost heavy for colleagues; I am absent as a meeting coordinator. I have been on the other side, all these moments when I walk away are difficult to understand, you are there in the spot. The question is what do colleagues expect next? It is a dichotomy: I am also an educator, when I can I work in the field, but I also have the other aspect to pursue. There is confusion, I do things on Sunday mornings when it is calm, otherwise 30 people enter the office…
(C4): Yes, yes, … How far are we available? If I am available, you pay me, give me a mobile phone….colleagues can have a certain freedom of action….. in the obvious gravity that I want to be called, but in general I am not the supreme leader, there are things they can handle they. I AM LIKE THEM, an educator. I do and act as coordinator, but besides what do my colleagues expect from me, what do I expect from them? Coordinator is a new figure… sometimes it passes that you are the supreme leader.
(C5): It depends on what you want to pass….. welcome when the operators act, I delegate a lot.
(C3): They do it to make me participate. But chat, social … you are inside your work 24 h a day…. is that right?
(C1): When it works everything is fine, but if something doesn’t work, look at what the coordinator did not do, following the job.

Coordinators experiment with confusion and strong social exposition, without having a defined perimeter of their role. It is difficult to find in the job description alone the meaning and limits of what one is called to be and do within a complex organization. If the job description arises, therefore, as necessary but not sufficient, an adequate ability to manage intersections and mobility must be increasingly exercised. The training setting gives the possibility to exercise liminal practices of reflexive thought, recognition, and experimentation of common events and situations, thematizing the reciprocal acknowledged boundaries, and plural expectations related to their role (the “part to be played”), and the function (conscious and transversal attention to various areas). The calibration and balance of time and space to be dedicated to different mandates, as well as the relationship with colleagues, are also strongly linked to the theme of delegation (or non-delegation) and the theme of physical and “virtual” availability.

The second plot is about to *manage* “*new entries*”*:* the evolution of professions and the differences between the professional families can create a feeling of disorientation with respect to the presence of new practitioners who meet the work and organizational reality of the center. The perception of new recruits as inflexible and unable to accommodate the reality of the chosen profession creates gaps and distances that can make it difficult to support a reconfiguration of one’s professionalism, avoiding the risk to escape from the organization.

In this regard, the conversational excerpt below (related to the fifth meeting after the acceptance of the role) sheds light on some routed in practice aspects:

(C6): Who comes to us? trainees, some who have yet to decide, others who have already decided. There is a time for the presentation of the structure and ways of working that precedes our interfacing with the person who arrives. Fascinating and showing that this is a good place to work is one thing, the first worries me, finding people who come to see first, especially educators.
(C5): I bring you my modality, not extrapolated from books, created *in itinere*. What is meant by new? When is one no longer so and walking alone? Over the years the center has changed its position compared to the new one: first educators came in because they needed to work, no need to create a charm; now several people pass by to whom an attractive product needs to be offered. Some people who arrived did volunteer days, then ran away. The personnel management asked us to welcome, propose work as if it can offer immediate satisfaction. I am asked to sell a product. I try to mediate. I ask for expectations upon arrival (3 days of volunteering, then people decide whether to stop) and if they expect to work in a group. A very simple interview. Then I explain how we are organized, the day, the type of guests, but without going into detail, superficial because the person could leave immediately. I tell them to observe the dynamics and interactions of the group (patients, operators,….), let them experience it.
(C8): Fear plays a lot on evaluation…
(C5): Then they are taken by the patients, I recommend that they read files and propose to ask questions if not clear and to copy rules and group dynamics. Not to take too much initiative or too little, a balanced way. After 2–3 weeks, return to the person and request their feedback. There comes the flaw, maybe I’ll do another one later, but the method is not complete. If you have to suggest. When is one no longer new? When can you manage the group in the absence of older educators? It happens that I solicit certain things and then the operator does not apply them, sometimes with the intervention of clinicians. The departure is similar with everyone, then each educator is different, and you have to adapt to his personality. I see in my method the limit of an effective continuity; it is lost when one is “inserted.” On two occasions, personal management asked me to make evaluations on a form…

The work of adapting to the context, integrating what has been studied with the actual life and functioning of the organization, is complex. The need to create spaces for knowledge and contact with new potential new entries (students, practitioners seeking a new job, junior professionals, etc.) to create internship opportunities that convey value and knowledge are some possible tools for promotion and visibility both for the organization, which must present itself as attractive and create interest and for the units, prime mediators of the impact between newcomers and the working environment. These spaces, inhabited by the coordinators together with other figures, can generate paths and activities, products and materials that take into account the challenges, the beauty of the work, the differences between professional families, and which are the result of a concrete work of weaving and communication.

A third plot refers to *bridging different professional/organizational cultures and families*: coping up with a progressive rooted in practice experience as coordinators, they become aware of the dynamics and needs within a complex structure that requires the group to be “open” and work for integration. Dynamics that can generate sustainable balances or lead to the loss of boundaries even between the professional families (nurses who “They act as educators,” director of a “factotum” structure). Prompting alliances, intersections, and goodwill can allow a more synergistic functioning in the absence of resources, but at the same time it can generate a lack of clarity of roles that produces further confusion. Finding moments of meeting between the individual professionals to talk about the professional family and their identity and contribution becomes a decisive element in reworking and regenerating a professional culture identified despite its flexibility, as the excerpts below (related to the sixth meeting) point out:

(C9): There is a demand for flexibility and porosity that unites us, but many guys (he means the operators, “young people trained”) who came to me ran away. We asked ourselves questions. I thought I had Cracco’s (a famous Italian cook) kitchen. Perhaps they have particular training. It happened to me that they asked me for a recipe for each situation and one is disoriented with respect to the expectations these guys have on our work. We ask them to integrate in everything and this has made them explode and we with them. There is the turnover of people who try to train and support from all points of view…. this youth that arrives, what we do to them?
(C11): A new figure of psychiatric rehabilitation therapist arrived and she thought she was working one by one, one patient at a time, not being catapulted into a group of psychiatrists. When we started, there wasn’t all this protectionism. We put ourselves in this way, for protection, let’s make him feel at ease…. there is everything, but then the reality is this, of pain, suffering, physical and psychological risks…. but this is part of the game! maybe it’s a training problem? I have seen different availabilities over the years.
(C5): An educator expects certain things, a therapist quite other.
(C10): We lack educators and are replaced with occupational therapists and other figures that are quite distant in terms of training…
(C6): We all come together for each module, to ensure communication, information and training, to help each other in everyday life between the observer, educators and nurses. Nurses do not come often from the central building, and they are new, they need a job of socialization and to help them understand the difficulties of the users.

A fourth relevant plot is about *facing contradictions while seeking for a good work*: in the final meetings, the fatigue and hindrances related to tackle the complexity, the ambiguities, and the intertwined tangle of the texture of coordinators practice arise. Problems and difficulties of the practice are soliciting the professional identity, as well as the troubles of reshaping own’s professional identity meet and draw unfolding practices. The conversational excerpts below (seventh meeting) are emblematic:

(C7): I can’t keep banging against the doors, there are crystallized situations that remain there…. it’s not that the kettle from 90° goes to 50, it goes to 120 but it’s in the pressure cooker, everything doesn’t calm down if no decisions are made…. how can I, coordinator with “all this power,” help what happens…. if I tell you that someone is sick, how can you tell me “Not that one because he knows xy, I don’t want that….?” Even benefits, in my group there are people who give even exaggerated availability and I don’t give benefits. There’s the smart one who stays home sick. I have people who automatically change turnover to work together. It’s been made clear that it’s not okay, but they do their own bullshit. As coordinator I cannot intervene because they tell me “Flexibility….,” but this is a big limitation. And the directional board that tells me: “It’s a big problem. Let’s move on to something else …”
(C4): There are colleagues who make the sign of the cross when they work in a group with the coordinator because they work with one less…
(C7): I have to ask someone to please come and I have no power to change hours if there is not an emergency. You are in limbo, some you cannot touch them, some things you cannot do…. should be healed. With C.4 I have dealt with a lot…. he represents the vanguard of the proposal that the Center is giving. His schedule is separate from that of the others and he tells me: “I think this is the point of arrival.” Which means taking on even more commitments. I can go directly to the source, to the social worker, avoid seven bureaucratic steps because I have it in the job. Obviously he takes up time and he is not working among the flowers, but he is ahead. and in my opinion this mode is closer to the idea of coordinator than in the other units it has not been done. We clash with resources, limitations. but to bring even just a part of our timetable beyond.

Contradictions challenge the unfolding process of shaping the coordinators’ identity (eight meeting):

(C6): Ours is not a good job, we are everyday all day in contact with suffering. I like it, but it’s not a good job, maybe the good job is the ice cream maker. There is a way to do it better or in a certain way. Propose in groups a quality of work and create something that is beautiful…. this makes it easier to ask for more from colleagues, one extraordinary or other. Quality depends on many things, tools, interface with the territory. The organization must provide tools and I must motivate the organization to provide them. If I do a cooking workshop with the kids, I can’t get a camp stove.
(C12):… mmhhh…yes, yes, but what does make a job a beautiful one? Why you don’t apply as ice cream maker? (all people laughing).
(C1): …of course we face a lot of troubles…really….but I think that we are living our life annuity facing all this magmatic material…

Doubtless, a job in contact with suffering and inhabiting the contradiction of offering a quality service in the face of limited resources is a challenge. You need an identification with the work object and with a sense to be given to one’s action, as well as an awareness of the organization around the practitioner and his/her relationship with the work object as a “good job” (Gardner et al., 2001). It is a competent job, which fully exploits individual abilities, generates satisfaction and value for individual and for others, it is a job whose object is developed and experienced as pleasurable and sustainable. Creating personal and collective sustainability through interpretations of the possibilities, synergies, and usable resources is part of a “good job,” capable of responding effectively, and satisfactorily to the real and complex needs of the professional.

## Discussion and Conclusion

The described plots highlight how the liminal space of the training setting can develop both a reflexive and an imaginative work about experiences of identity separation vs. continuity, of ambivalence and insecurity. At stake is the possibility to share meanings with respect to one’s own experience as a possible outcome (neither predictable nor automatic) of moments of exchange and comparison. The liminal space becomes the scene of concrete events and situations, through which the coordinators give meaning and shape their contexts and practices. The idea of organization as a social artifact, as an arena of negotiated *(dis)order*, appears concrete and tangible through the flow of voices, languages, actions, tacit knowledge used to interpret and shape one’s activity, and organizational role.

Indeed, the implementation of a process of hybridization, required by the current organizational scenarios, characterized by uncertainty, rapid evolution, complexity, and ambiguity (Bennett and Lemoine, 2014), entails a progressive transition of personal, professional, and social identity. People are asked to face the challenging activation of a *nomadic movement* because one moves from one point to another, one unit to another, one work object to several, often divergent objects, according to a trajectory only partially pre-definable and constantly exposed to turbulence and uncertainties that require visual navigation, with continuous adjustments and adaptations.

All the organizational actors are called to an articulated movement that requires keeping up with mental (representations, expectations, and orientations), relational (exchanges, relationships, and integrations), and corporeal (fatigue, resistance, and rhythm) aspects. A movement related on knowing when to speed up and when to slow down, how to adjust the many degrees of speed; movement as an often acrobatic search for sustainable balances for oneself and for others, to be built and implemented in a creative way.

By analyzing the emergent plots in the liminal training setting and the liminality on which it is embedded and routed, it is possible to highlight the four relevant movements that are closely intertwined and give rise to the plural manifestations of professional hybridization, necessary to stay within adhocratic organizations capable of governing the unexpected in the concrete work contexts in which one is called to operate.

The first movement is internal, relating to personal investment, expectations, taking a choice on how to answer the question “why do I do the work I do?”

The second movement is operative, concerning the professional identification with a work object that is transformed and can take on multiple tasks, plural levels of work, different and often contradictory objectives to be faced.

The third movement is reflexive, connected to the need to transform being absorbed into specific deliberate tasks and efforts, thinking critically about one’s position and actions, and acquiring thematic intentionality (Yanow and Tsoukas, 2009) about what to do be made.

The fourth movement is institutional and concerns the construction of a sufficiently good alliance with the horizontal and vertical stakeholders with respect to the possibility of activating organizational work (Cecchinato, 2019), through which the framework of meaning and recognition of one’s work, the things to do and how to do them, the power relations, and the division of labor and the existing possibilities for development are socially negotiated.

Undertaking and interpreting these movements mean assuming a *nomadic vision*, dealing with a work object that reconfigures itself rapidly and with constantly changing scenarios; it implies living in a borderline position, like tightrope walkers who go through multiple tensions and interpret a precise representation of their role.

Recognizing these movements becomes part of the responsibility of those who are called to exercise managerial and social skills within organizations, feeding articulation processes related to the generation, maintenance, and change of agreements, actions, regulations between people and organizational units functional to achieve common objectives and goals. The training setting as a liminal space can trigger and develop connections between different parts and components, weaving and stabilizing relationships to share skills, resources, and knowledge (Scaratti et al., 2017).

The activation of such dynamics is not taken for granted and leads to the first research question related to the pivotal features that characterize a training setting as a liminal space. We can underline its peculiar configuration as an *organizational narrative space* that requires peculiar hallmarks and conditions, which ensure its sustainability and effective practicability:

- The acquisition of dimensions of *trust and mutual recognition* (in terms of value and credit attributed) constitutes a preliminary variable for respectful and interested listening, both by the researcher/consultant toward the organizational actors and their context and by various stakeholders among themselves. The reciprocal exposition of one’s own narratives is neither obvious nor automatic and requires the activation of appropriate listening and protection situations.

- An adequate *regulation of proximity/distance* with respect to the events that occur and their implications for organizational and working practices. This is an indispensable element to convey interest and mobilization toward constructive and relevant outcomes for the participants involved. This does mean welcoming the narrative fragments already present and facing their affective implications (Cunliffe et al., 2019), proposing ideas around which circulating readings and reconstructions could converge and find acceptance. This makes it possible to identify the organizational processes and real problems present in the common field of experience, opening concrete spaces for a re-reading of one’s own interpretations, and configurations of meaning.

- A proper use of a *variety of linguistic and discursive accounts* (tales, stories, conversations, and written documents) enabling multiple levels of involvement (that of the researcher/consultant, various actors with their individual stories, the organizational narrative reread and restarted, and the narratives woven by the practitioners with their organizational units to which they belong) and enhancing a sort of dialogic and narrative texture, which feeds stories in turn generators of renewed narrations.

- An *institutional mandate* to address concrete situations and organizational events through which the framework of meaning and recognition of one’s work is socialized and negotiated, as well as the things to do and how to do them, the power relations, the division of labor, and the existing development possibilities.

In relation to the second research question, about the possible plots of professional hybridization that a training group as a liminal space can trigger and develop, we highlighted four emerging wefts: dwelling the boundaries between the roles, managing “new entries,” bridging different professional/organizational cultures and families, and facing contradictions while seeking for good work. All of them are triggered by in-between liminality spaces as the described training setting, through which a personal and social identity accomplishment is improved and reflexively thought.

The need to deal with ambivalences related to the complexity of the profession and the managed role has emerged in a more focused way on perspectives that offer relief in the face of problems as they arise. The opportunity of achieving finer adjustments and long-range textures, which creates a sustainable level of programming and adjustment, has been progressively thematized.

The possibility to conceive professional and organizational identity as blurred and continuously reshaped, going beyond a formal job description of a specific position in a hierarchical structure, allows experiencing an actionable and a transitory place in which uncertainty, vulnerability, and insecurity can become a trigger for transformational changes.

In conclusion, the concept of liminality, associated with the metaphor of social limbo, with its potential expression of generative evolutions, sounds suitable and good to be applied to professional and organizational landscapes, specifically those entailing interactive, collective, and social processes.

## Data Availability Statement

The raw data supporting the conclusions of this article will be made available by the authors, without undue reservation.

## Ethics Statement

The studies involving human participants were reviewed and approved by the Department of Human and Social Sciences, University of Bergamo. The patients/participants provided their written informed consent to participate in this study.

## Author Contributions

GS and SI participated to the action research and described and wrote the theoretical part of the manuscript. EF was involved in analyzing, selecting, and writing the empirical data to be used in the excerpts. All authors contributed to the article and approved the submitted version.

## Conflict of Interest

The authors declare that the research was conducted in the absence of any commercial or financial relationships that could be construed as a potential conflict of interest.

## Publisher’s Note

All claims expressed in this article are solely those of the authors and do not necessarily represent those of their affiliated organizations, or those of the publisher, the editors and the reviewers. Any product that may be evaluated in this article, or claim that may be made by its manufacturer, is not guaranteed or endorsed by the publisher.
